# 700. Risk Factors and Molecular Epidemiology of Acute and Chronic Norovirus Infection at a Large Tertiary Care Cancer Center

**DOI:** 10.1093/ofid/ofab466.897

**Published:** 2021-12-04

**Authors:** Divya S Kondapi, Sasirekha Ramani, Adilene Olvera, Robert L Atmar, Mary Estes, Pablo C Okhuysen

**Affiliations:** 1 Baylor College of Medicine, Coral Gables, FL; 2 The University of Texas MD Anderson Cancer Center, Houston, TX

## Abstract

**Background:**

Norovirus (NoV) is the leading cause of viral diarrhea in patients with cancer. In this study, we describe risk factors associated with acute and chronic NoV infection in this patient population.

**Methods:**

We identified 132 patients with NoV diarrhea (using stool RT PCR) between 2016-2020 at University of Texas MD Anderson Cancer Center (MDACC). Patient data, including demographics, clinical characteristics, NoV treatments, and complications were retrospectively extracted from charts. Stool samples were analyzed for NoV genogroups and genotypes. We compared characteristics and outcomes of patients with acute diarrhea (< 14day; AD) versus chronic diarrhea ( >14day or recurrences within 12 weeks; CD) and analyzed the data using Pearson Chi square or Fisher’s exact for categorical variables and Wilcoxon rank-sum test for continuous variables.

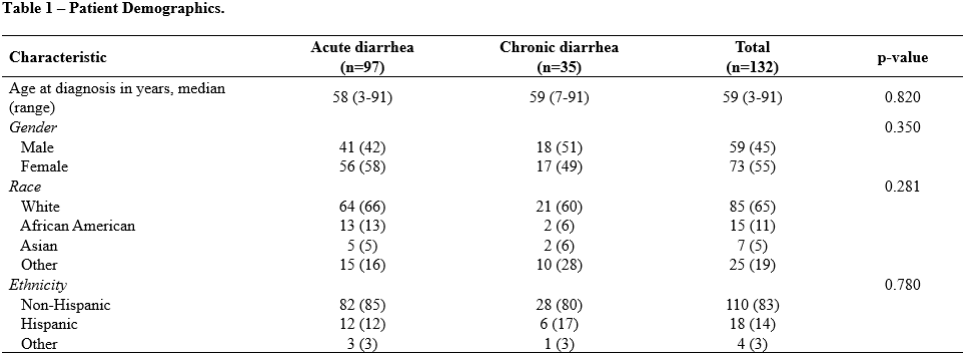

**Results:**

Of 132 patients identified, 124 had an underlying cancer (39 solid tumor, 85 hematological malignancies, Table 1). On univariate analysis, CD patients were more likely to have a hematological malignancy (p=0.002), be a hematopoietic stem cell recipient (p= 0.013), have a history of gastrointestinal graft versus host disease (p= 0.011), or have received immunosuppressants or steroids in the 90 days before diarrhea onset (p=0.001, Table 2). CD patients had significantly lower white blood cell counts (p=0.038), absolute neutrophil counts (p=0.049), IgG levels (p= 0.001), and serum albumin levels (p=0.002) at the time of NoV diagnosis (Table 3). Patients with CD more often received symptomatic or NoV targeting treatment, including anti-diarrheal (p=0.005), nitazoxanide (p< 0.001), intravenous immune globulin (p=0.017), and oral IgG (p=0.042). CD patients more often had diarrheal recurrence in the first 4 weeks (p=0.001) or the second month (p< 0.001) after initial diagnosis and needed enteral or parenteral nutrition (p=0.004). We genotyped NoV in 67 patients (Figure 1), resulting in identification of the following genogroups: GI (n=9, 13%), GII.4 (n=23, 34%), and other types of GII (n=35, 52%). Genotype diversity was higher in patients with CD than AD (Figure 1).

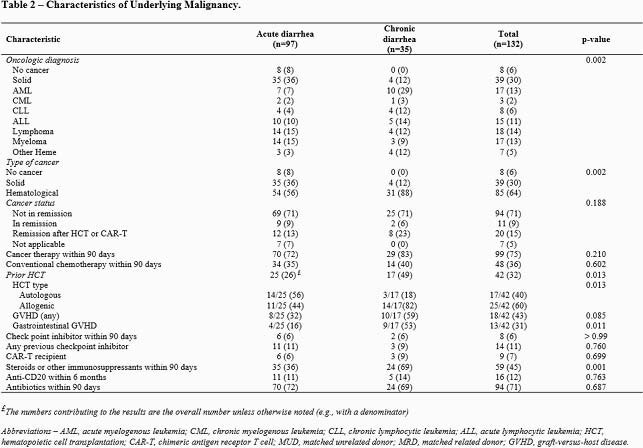

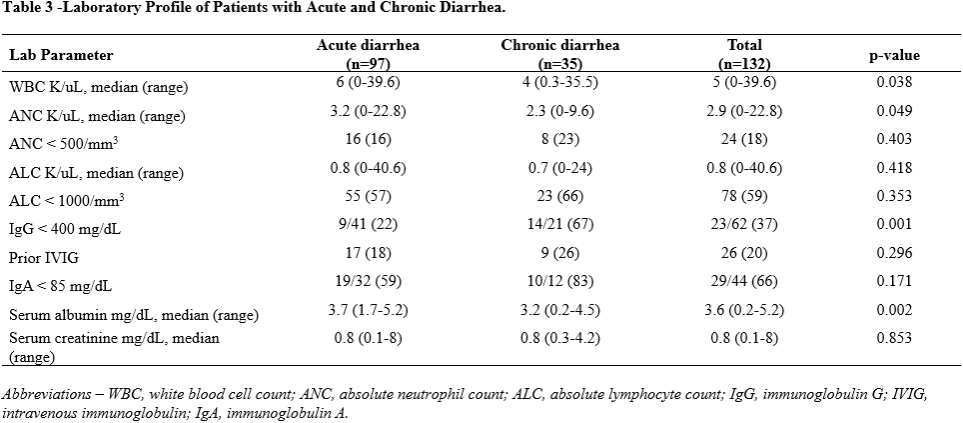

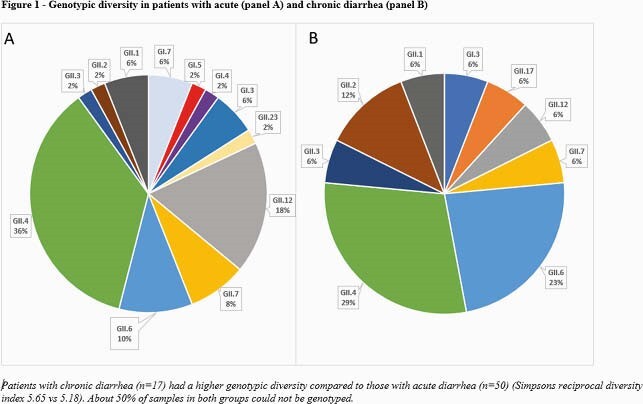

**Conclusion:**

In patients with cancer, CD from NoV is associated with severe immunosuppression, is refractory to therapy and can be caused by a variety of NoV genotypes/genogroups.

**Disclosures:**

**Robert L. Atmar, MD**, **Takeda Vaccines, Inc.** (Grant/Research Support) **Mary Estes, PhD**, **Takeda Vaccines** (Consultant, Grant/Research Support) **Pablo C. Okhuysen, MD, FACP, FIDSA**, **Deinove Pharmaceuticals** (Grant/Research Support)**Ferring Pharmaceuticals** (Consultant)**Melinta Therapeutics** (Grant/Research Support)**Merck & Co.** (Grant/Research Support)**Napo Pharmaceuticals** (Consultant, Scientific Research Study Investigator, Research Grant or Support)**Singulex** (Consultant)**Summit Therapeutics** (Grant/Research Support)

